# Role of Predatory Mites in Persistent Nonoccupational Allergic Rhinitis

**DOI:** 10.1155/2016/5782317

**Published:** 2016-06-28

**Authors:** Paloma Poza Guedes, Inmaculada Sánchez Machín, Víctor Matheu, Víctor Iraola, Ruperto González Pérez

**Affiliations:** ^1^Allergy Department, Hospital del Tórax, HUNS La Candelaria, 38320 Santa Cruz de Tenerife, Spain; ^2^Department of Clinical Sciences-Division IV, Lund University, 22100 Lund, Sweden; ^3^Laboratorios LETI, 28760 Madrid, Spain

## Abstract

Mites can sensitize and induce atopic disease in predisposed individuals and are an important deteriorating factor in patients with allergic rhinitis, asthma, and atopic dermatitis. Although Pyroglyphidae mites have been extensively studied, very scarce reports are available on Cheyletidae spp. especially regarding human respiratory pathology. The main objective of the present study is to investigate the clinical role of this predator mite (*Cheyletus eruditus*) as a respiratory antigen in a selected sensitized human population. Fifty-two adult patients were recruited from the outpatient allergy clinic to assess their eligibility for the study. The thirty-seven subjects with persistent allergic rhinitis (PAR) who fulfilled the ARIA criteria had a positive IgE response confirmed by skin prick test (SPT) to* C. eruditus*. Only those individuals (37/47) with a positive SPT to *C. eruditus* showed a positive nasal provocation test (NPT), while 10 patients with nonallergic mild-to-moderate persistent rhinitis,* control group*, had a negative NPT with *C. eruditus*. The present paper describes a new role for the predator mite* Cheyletus eruditus* as a respiratory allergen in a selected subset of patients in a subtropical environment afflicted with persistent nonoccupational allergic rhinitis.

## 1. Introduction

The house dust mites (HDM) are described as common aeroallergens, colonizing beds, sofas, carpets, and any woven material [1, 2]. HDM sensitize and induce atopic disease in predisposed individuals and are an important deteriorating factor in patients with allergic rhinitis, asthma, and atopic dermatitis [3–5]. Dust mites are also the main cause for respiratory allergies in the Canary Islands, a subtropical region offshore Spain. As described in some other areas of the world with similar weather characteristics* pyroglyphid* and* nonpyroglyphid* mites can be found not only in storage facilities but also in the domestic environment [6, 7]. A report from our work group found that the predator mite* Cheyletus *spp. was unexpectedly frequent in house dust samples collected in our location, Tenerife [8]. Although Pyroglyphidae mites have been extensively studied, very scarce reports are available on Cheyletidae spp. especially regarding human respiratory pathology. The main objective of the present study is to investigate the clinical role of* Cheyletus eruditus* as a respiratory antigen in a selected sensitized human population.

## 2. Material and Methods

### 2.1. Subjects

We recruited adult patients consecutively with a clinically staged diagnosis of mild-to-moderate persistent allergic rhinitis according to the ARIA guidelines [9, 10] from the Allergy Outpatient office. Only patients with a positive skin prick test (SPT) referring to the* Cheyletus eruditus* extract were included in the case study group. The control group consisted of subjects presenting with mild-to-moderate persistent nonallergic rhinitis (i.e., subjects with negative SPT and/or serum specific IgE to common airborne allergens including* Cheyletus eruditus*). Pregnant and breast-feeding women were excluded. The study was approved by the Ethical Committee of the hospital and informed consent was signed by all participants.

### 2.2. Allergen Extract

Mite bodies of a* C. eruditus* culture were carefully separated from the culture medium with forceps. The mite bodies were extracted (1 : 10 wt/vol) in PBS 0.01 M (pH 7.4) overnight at 4°C under continuous magnetic stirring. Afterwards, the extract was centrifuged, the supernatant separated, and sterile-filtered and freeze-dried. The protein content of the extract was evaluated by the Lowry-Biuret method (Sigma, Bedford, MA, USA), using bovine serum albumin as standard. The protein content of* C. eruditus* extract was 125.2 mg/mL. The determination of specific IgE to* C. eruditus* was performed by LETI. The determination was done by ELISA. Briefly, 20 mg/mL of the protein extract was dissolved in carbonate/bicarbonate buffer, pH 9.6, and plastic microtiter plates were coated in the dissolved extract (Immulon IV; Dynex Technologies, Chantilly, VA). Each serum sample was diluted to 1 : 2 vol/vol in 0.01 M PBS and incubated (100 *μ*L) for 2 hours in the wells. The plates were washed and incubated for 2 hours in anti-human IgE conjugated with peroxidase. After 5 washes, the reaction was developed for 30 minutes and then stopped with sulfuric acid 1 N. Three individual serum samples from nonallergic patients were used as negative controls. Optical densities (ODs) 4 times the mean value of the negative controls or greater were considered positive.

### 2.3. Skin Prick Test and Nasal Provocation Test

Skin prick tests were performed with an extract containing 10 mg of freeze-dried material of* Cheyletus eruditus* (0.05 mg/mL) and standardized extracts for the routine airborne allergens present in our area (*D. pteronyssinus*,* D. farinae*,* Blomia tropicalis*, cat/dog dander,* Alternaria alternata*,* Artemisia vulgaris*,* Parietaria judaica*, and grass pollen; Laboratorios LETI, Madrid, Spain). Histamine and saline were used as positive and negative controls. Following everyday practice, antihistamines were withdrawn a week prior to the SPT. The wheals were read after 20 minutes and those diameters greater than 3 mm were regarded as positive.

The measurement of the clinical response to* Cheyletus eruditus* was assessed by a specific nasal provocation test (NPT). A solution of* Cheyletus eruditus* was diluted to 0.1 mg/mL w/v with saline (0.9%) to obtain dilutions at 1/10, 1/100, and 1/1000 mg/mL for each subject. The doses of topical (nasal and/or bronchial) steroids, antihistamines, and nasal vasoconstrictors were discontinued or minimized 2 weeks before the nasal challenge. The NPT began every morning (8:30 a.m.) to avoid possible nasal cycle influence. After a resting period of 30 minutes, both acoustic rhinometry (Rhinoscan, Interacoustics A/S, Denmark) and forced bronchial spirometry (Sibelmed, Barcelona, Spain) were performed to measure the basal nasal minimal cross-sectional area (MCA) [11, 12] and nasal volume 2–5.5 cm^3^ (Vol.) besides lung function forced expired volume in one second (FEV1). The nasal challenge was considered positive with a significant drop (above 25%) over the basal nasal volume. Saline solution was previously sprayed into both nostrils to rule out nasal hyperresponsiveness (NHR). Increasing doses of* Cheyletus eruditus* were sprayed unilaterally in the same nostril while rhinometric,* every 15 min*, and spirometric,* every 30 min*, measures were taken after every nasal application. A clinical score was also measured throughout the nasal challenge and regarded as positive above five points [13, 14]. The significance of changes was assessed by Mann-Whitney *U* test. Statistical significance was assumed at *p* values ≤ 0.05.

## 3. Results

### 3.1. Patients, Sera and Skin Prick Test

Forty-seven adult patients (Table 1) were recruited from the outpatient allergy clinic to assess their eligibility for the study. The severity of the persistent allergic rhinitis (PAR) was assessed using the Allergic Rhinitis and its Impact on Asthma (ARIA) guidelines. The thirty-seven subjects (23 females and 14 males, median age 30.5 y.o.) with PAR who fulfilled the ARIA criteria—*conforming to the active study group*—had a positive skin prick test (SPT) to* C. eruditus*. Only two out of the thirty-seven subjects had a single sensitization to* C. eruditus* while the rest of the individuals were also allergic to different mites—*Dermatophagoides* spp. and/or* Blomia tropicalis*. The control group consisted of 10 patients with nonallergic persistent rhinitis (i.e., subjects with a negative SPT and/or serum specific IgE to common airborne allergens including* Cheyletus eruditus* (control group: 6 females and 4 males median age 29.0 y.o.)).

### 3.2. Specific Nasal Challenge and Acoustic Rhinometry

All forty-seven patients included in the present study underwent a specific NPT with* C. eruditus*. Only those subjects (27/37) with a positive SPT to* C. eruditus* showed a positive NPT with the same specific mite while 10 patients with nonallergic mild-to-moderate persistent rhinitis—*control group*—had a negative NPT with* C. eruditus*. All positive nasal responses were found in the same nostril (*ipsilateral*) while no contralateral nasal responses alone were obtained. Nasal nonspecific reactivity was excluded by means of a saline solution sprayed into the nose prior to each specific NPT in both study groups. Mean MCA and VOL were significantly (*p* < 0.05) decreased only in those patients sensitized to* C. eruditus*. No differences were found in basal FEV1 in both groups and no changes were found in those FEV1 obtained throughout the NPT and at the end of the observation period in both groups. A positive NPT clinical score was found in the active group but not in the control subjects. One patient of the active group had a clinical score lower than 5 (3 points); a subsequent conjunctival provocation test was performed with a* C. eruditus* extract ending with a positive result at 1/10 dilution. All patients with a positive NPT were subsequently treated with a single oral antihistamine (cetirizine 5 mg) and topical nasal oxymetazoline. No inhaled beta-agonist, systemic steroids, or adrenaline was needed at any time and no admissions to hospital were required. Although home peak nasal inspiratory flow (PNIF) measurements were not assessed, no late phase reactions were reported by the patients.

## 4. Discussion

Pyroglyphidae mites, a family of house dust mites, are the most relevant antigens causing persistent respiratory allergy worldwide [3, 4] and their major allergens have been extensively studied [15]. There is an ongoing progress in the understanding of the human IgE response to nonpyroglyphid storage mites and the allergenic cross-reactivity with other mite species [16–19]. Studies from several countries have shown relevant IgE-mediated allergy in rural populations and that storage mites are major allergens. Since these mites are found in homes, especially in regions with damp housing conditions, urban populations are at risk of becoming sensitized [20].

*Cheyletus eruditus* is a predatory mite that commonly lives in bulk food stores such as granaries. It is also often found in animal feed, bedding, house dust, poultry litter, and mammal and bird nests. In the subtropical Canary Islands, dust mites account for the most common cause for respiratory allergies. Interestingly, a high prevalence (51%) of* Cheyletus eruditus* was obtained in a 2004 study of house dust samples from mattresses and carpets from this area (Tenerife, Spain) [8]. The Cheyletidae have been associated with parasitism in birds and mammals, but also in human dermatitis [21]. Yoshikawa [22] confirmed that these mites were able to bite and induce cutaneous histopathological changes in human skin. In fact, the presence of human corporal fluid was confirmed only in the stomach of Cheyletidae mites but not in other family species, like* Dermatophagoides farinae*,* Dermatophagoides pteronyssinus*, and* Tyrophagus putrescentiae*. The relevance of including different dust mites in the routine allergy diagnosis work-up has been previously published and several studies demonstrate the complexity of the immunologic responses to different mite species [23–25].

We initially described between 2008 and 2012 [26, 27] IgE-human sensitization and the clinical features in a selected population of 13 atopic patients sensitized to* Cheyletus eruditus*. Those data lead us to investigate the putative role of this “new allergen” in causing respiratory,* that is*,* allergic rhinitis*, symptoms. In the current study, a larger population of 42 cases showed IgE sensitization to* Cheyletus eruditus* having obtained a positive specific nasal response in all those 27 patients with persistent allergic rhinitis when exposed to this predatory mite. Despite the small size of the present sample a high degree of immunological and clinical reactivity with* Dermatophagoides* spp. and* Blomia tropicalis* was expected and confirmed by in vivo testing. As this group of patients are exposed to both* pyroglyphid* and* nonpyroglyphid* mites, further investigations are warranted to discriminate between IgE cross-reactivity and dual-sensitization. Regrettably, we only found 2 patients who were selectively allergic to* Cheyletus eruditus* and not to* Dermatophagoides* spp. or* Blomia tropicalis*. The current NPT with* C. eruditus* proved to be safe in all patients, as immediate symptoms were only locally (nasal) elicited and no late clinical reactions were recorded. Predatory mites are commonly used as biological pesticides worldwide for control of spider mites and other pests in greenhouses. In 1999, a study in bell pepper growers described the* Amblyseius cucumeris* as a relevant source of occupational allergy with 23% of the population having positive skin prick test reactions [28]. A subsequent work confirmed that* Amblyseius cucumeris* has both species-specific antigens and common antigens that are cross-reacting with* D. Pteronyssinus* [29]. Interestingly, the predatory mites* Amblyseius californicus* and* Amblyseius cucumeris* have also been described as a cause of occupational asthma [30].

The present paper describes the potential role as a respiratory allergen for the predator mite* Cheyletus eruditus* in a selected subset of patients in a subtropical environment who had symptoms of persistent allergic rhinitis. As allergic respiratory patients may show different immunologic patterns of dust mite sensitization, the identification of new allergens is critical to achieve an accurate diagnosis and relevant treatment of their allergic conditions. Our findings suggest that the predatory mite* Cheyletus* spp. may be considered a relevant airborne allergen not only in occupational respiratory medicine but also in persistent nonoccupational allergic rhinitis especially in those populations living in humid tropical and/or subtropical climates.

## Figures and Tables

**Table 1:** Patient data and specific nasal provocation test with *Cheyletus eruditus*.

Patients (*n* = 47)	Age	SPT *Cheyletus eruditus*	SPT *Dermatophagoides *spp.	SPT *Blomia tropicalis*	NPT *Cheyletus eruditus*	% variation in MCA after NPT	% variation in VOL after NPT	*Cheyletus eruditus* dilution (NPT)	Clinical score after NPT
1	28	+	+	+	+	31.15	42.38	1 : 10	7
2	38	+	−	−	+	34.55	38.51	1 : 10	6
3	20	+	+	−	+	27.28	40.58	1 : 100	8
4	48	+	+	−	+	64.29	72.4	1 : 10	9
5	29	+	+	−	+	8.02	31.7	1 : 10	6
6	21	+	+	−	+	51.43	62.56	1 : 100	6
7	28	+	+	+	+	70.59	47.11	1 : 100	9
8	21	+	+	+	+	69.24	86.03	1 : 10	8
9	31	+	+	+	+	37.84	38.55	1 : 10	9
10	23	+	−	−	+	41.03	43.69	1 : 100	7
11	20	+	+	−	+	33.34	5.69	1 : 1	3
12	31	+	+	−	+	7.41	43.01	1 : 10	7
13	21	+	+	−	+	27.91	40.8	1 : 10	8
14	24	+	+	+	+	50.77	56.91	1 : 10	6
15	26	+	+	+	+	40.68	31.92	1 : 10	9
16	34	+	+	−	+	96.97	94.43	1 : 1000	9
17	45	+	+	−	+	33.34	86.66	1 : 10	8
18	36	+	+	+	+	29.4	13.14	1 : 100	8
19	23	+	+	−	+	29.04	32.18	1 : 10	9
20	25	+	+	−	+	52.0	52.15	1 : 10	10
21	34	+	+	−	+	25.0	45.57	1 : 10	7
22	31	+	+	+	+	47.3	42.79	1 : 100	7
23	32	+	+	−	+	34.15	18.08	1 : 10	6
24	31	+	+	−	+	20.38	7.04	1 : 1	8
25	30	+	+	−	+	27.66	42.48	1 : 10	9
26	51	+	+	+	+	40.0	52.58	1 : 10	6
27	35	+	+	−	+	38.58	22.4	1 : 10	8
28	23	+	+	−	+	36.85	40.45	1 : 10	9
29	24	+	+	−	+	48.44	40.31	1 : 10	6
30	25	+	+	−	+	20.46	26.0	1 : 100	7
31	37	+	+	+	+	7.41	37.78	1 : 100	8
32	18	+	+	−	+	43.4	38.89	1 : 10	10
33	18	+	+	−	+	18.75	21.81	1 : 10	9
34	33	+	+	+	+	37.5	35.3	1 : 1000	8
35	21	+	+	+	+	48.94	18.01	1 : 100	8
36	31	+	+	−	+	17.8	31.27	1 : 100	6
37	18	+	+	+	+	25.93	24.11	1 : 10	7
38	29	−	−	−	−	0.71	4.73	1 : 1	2
39	63	−	−	−	−	0.0	3.41	1 : 1	1
40	22	−	−	−	−	0.36	0.0	1 : 1	1
41	21	−	−	−	−	0.0	2.09	1 : 1	2
42	18	−	−	−	−	0.30	0.0	1 : 1	4
43	32	−	−	−	−	0.28	2.55	1 : 1	1
44	27	−	−	−	−	0.37	3.23	1 : 1	2
45	35	−	−	−	−	0.0	4.30	1 : 1	3
46	29	−	−	−	−	0.29	0.0	1 : 1	1
47	31	−	−	−	−	0.0	2.38	1 : 1	0

SPT: skin prick test.

MCA: minimal cross-sectional area.

Vol: nasal volume in cm^3^ from cm #2 to cm #5.5 (inflammation-susceptible mucosa) in the challenged nostril.

All 37 subjects included in the active study group were treated with oral cetirizine 5 mg and topical oxymetazoline after the positive nasal provocation test (NPT) to *Cheyletus eruditus*. No adrenaline or steroids were needed. The control group (10 subjects) required no medical treatment after the NPT in any case.
